# Automated and modular protein binder design with BinderFlow

**DOI:** 10.1371/journal.pcbi.1013747

**Published:** 2025-11-21

**Authors:** Nayim González-Rodríguez, Carlos Chacón-Sánchez, Oscar Llorca, Rafael Fernández-Leiro

**Affiliations:** Structural Biology Programme, Spanish National Cancer Research Centre (CNIO), Madrid, Spain; Shiraz University, IRAN, ISLAMIC REPUBLIC OF

## Abstract

Deep learning has revolutionised *de novo* protein design, with new models achieving unprecedented success in creating novel proteins with specific functions, including artificial protein binders. However, current workflows remain computationally demanding and challenging to operate without dedicated infrastructure and expertise. To overcome these limitations, we present BinderFlow, an open, structured, and parallelised pipeline that automates end-to-end protein binder design. Its batch-based architecture enables live monitoring of design campaigns, seamless coexistence with other GPU-intensive processes, and minimal user intervention. BinderFlow’s modular design facilitates the integration of new tools, allowing rapid adaptation to emerging methods. We demonstrate its utility by running automated design campaigns that rapidly generate diverse, high-confidence candidates suitable for experimental validation. To complement the pipeline, we developed BFmonitor, a web-based dashboard for real-time campaign monitoring, design evaluation, and hit selection. Together, BinderFlow and BFmonitor make generative protein design more accessible, scalable, and reproducible, streamlining both exploratory and production-level research. The software is freely available at https://github.com/cryoEM-CNIO/BinderFlow under the GNU LGPL v3.0 license.

## Author summary

The design of artificial proteins that specifically bind protein targets is a promising strategy for developing new therapeutics and research tools. However, current computational pipelines for binder design are complex to operate and rely on large-scale computing resources. Here, we present BinderFlow, a modular and parallelised workflow that simplifies *de novo* protein binder design. By dividing design campaigns into small, independent batches, BinderFlow allows efficient use of available GPUs, granular control of computational resources, and real-time monitoring. To fully take advantage of this architecture, we built BFmonitor, a web-based interface to visualise campaign metrics, evaluate design quality, and extract promising candidates for experimental validation in real time. We expect both BinderFlow and BFmonitor to make protein design more accessible, enabling researchers from diverse scientific backgrounds to engage directly in the design and refinement of *de novo* protein binders.

## Introduction

Proteins are complex biomolecules that perform many different functions, from catalysing chemical reactions to modulating regulatory networks through protein-protein interactions (PPIs). This functional versatility is mediated through the wide range of structures they adopt [1]. However, the structural space explored by natural proteins is limited, and thus the functions these proteins fulfil do not represent the complete range of potential tasks [2,3]. The possibility of creating proteins from scratch [4] with tailored functions [5] has made *de novo* protein design a major goal in molecular biology.

Sampling the protein-sequence space using biophysical methods quickly becomes computationally intractable due to its vast dimensions. The recent development of generative models provides an alternative to physics-based methods. These deep-learning models, trained on large datasets of proteins, can generate structures and sequences that fulfil a pre-specified function from scratch using a moderate amount of resources and time [6–9].

One of the many applications of artificial proteins is to bind to a specific surface of interest on a target protein, modulating its functions [10–13]. Traditionally, the production of such PPI regulators has required animal immunisation for antibody production or massive screening of chemical libraries. These methods require considerable experimental effort and present certain limitations, mainly: some interfaces lack defined pockets for small molecule targeting [14,15], antibody development involves high production and conservation costs [16], and some targets are not suitable for chemical screening [13,15]. Artificial proteins designed specifically to bind to a protein interface —binders from now on— overcome these problems, as they can be produced in *Escherichia coli*, feature extreme thermal stability, and exhibit high affinity and specificity towards their target [11,17,18]. Thus, the possibility to engineer binders in a targeted manner has both immediate therapeutic [12,19] and biotechnological implications [20,21].

**Fig 1:**
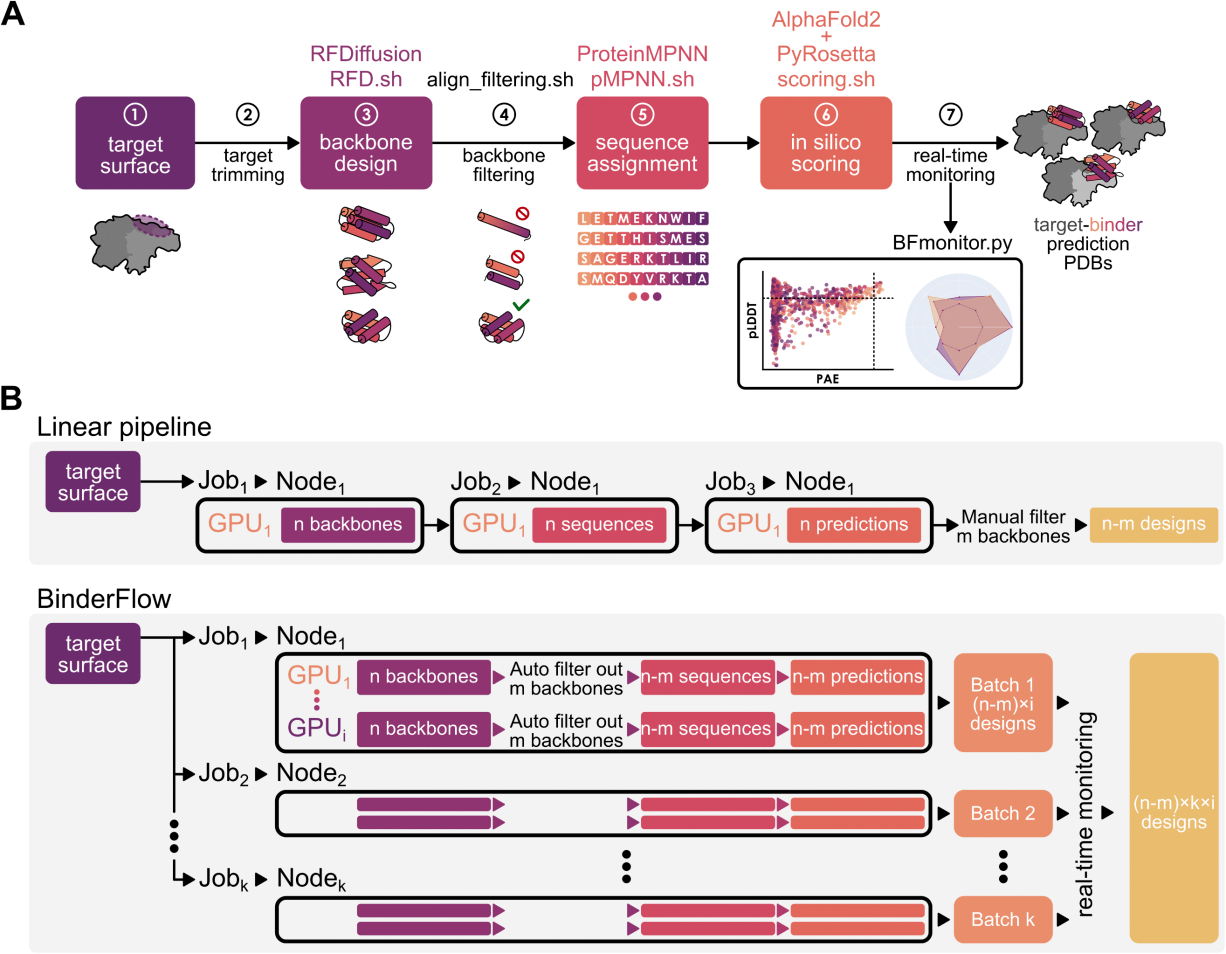
Binder design pipeline and BinderFlow architecture. **(A)** Schematic of the BinderFlow pipeline: 1. The user defines a hotspot in a surface of interest. 2. The target structure gets trimmed to increase computational efficiency. 3. RFD.sh produces protein backbones of a specified length complementary in shape to the target. 4. align_filtering.py filters out suboptimal backbones that might be problematic for expression, such as long helices or isolated hairpins. 5. pMPNN.sh assigns a sequence of amino acids to each backbone. 6. scoring.sh predicts the binder-target complex using AF2IG, collects the relevant AlphaFold2 scores and uses PyRosetta for measuring a set of relevant parameters for interactions. 7. The process is monitored in real time using the BFmonitor.py web-based tool. **(B)** Architecture of BinderFlow and comparison with a linear workflow. In a typical workflow each step of the pipeline requires manual handling of input and output files, and manual inspection to remove obvious suboptimal candidate backbones. Moreover, each step is run as an independent job, hampering parallelisation. Meanwhile, BinderFlow distributes batches of end-to-end predictions as independent jobs, facilitating parallelisation of instances across GPUs and HPC cluster nodes. It includes automatic filtering of suboptimal backbones and real-time monitoring of the process, allowing for stopping the campaign once a suitable number of hits is achieved.

A typical protein binder design project begins by selecting the region of interest on the target’s surface, thereby constraining the design space. Then, the process involves finding a backbone whose shape is complementary to the target surface and assigning a sequence of amino acids that folds into that backbone to establish intermolecular interactions with the target. The pipeline described by Watson *et al.* [22] has become the standard for *de novo* binder generation [18]. This pipeline uses Rosetta Fold Diffusion (RFD), a diffusion model that “denoises” previously unseen backbones from randomly distributed atoms [22], ProteinMPNN (pMPNN) to assign sequences to those backbones [23], and a modified version of AlphaFold2 (AF2IG) to assess the quality of the designs *in silico* [24–26], sequentially. AF2IG confidence metrics are then used to filter *in silico*-successful binders —hits from now on— which are then validated experimentally (Fig 1A).

Structural prediction scores are not infallible indicators of actual binding [27–29], so many designs still need to be screened and experimentally validated to find a successful one. Typically, tens or hundreds of protein designs are tested on plate-based assays for convenience and throughput, previously selected from computational campaigns that range from thousands to tens of thousands of candidates [10,12,17,22,30]. Such campaigns require computational resources that are often prohibitive for small or non-specialised laboratories. In addition, the default way to execute this pipeline [11,22,31] is to run each step sequentially as a single, large job in a linear fashion, i.e., generating thousands of backbones, then calculating the corresponding thousands of sequences, and finally evaluating thousands of potential binders using AF2IG. Notably, the *in silico* success rate for a given campaign, meaning the ratio between hits and total designs, varies widely [11,17,22] and cannot be estimated beforehand. This often results in inefficient campaigns, where the number of required designs is either overestimated, wasting limited computational resources, or underestimated, forcing the launch of a new set of jobs to increase the total size of the campaign.

Here, we report BinderFlow, a pipeline designed to make protein binder design more efficient and accessible, democratising *de novo* protein binder design for the wider scientific community. This approach divides a typical protein design project into arbitrarily sized batches, each performing backbone design, sequence assignment and candidate scoring in an automated workflow. By breaking the campaign into shorter tasks, BinderFlow enables more granular execution and live monitoring of progress. This structure also enables researchers to opportunistically use GPU resources —usually employed by unrelated workflows— for executing short-lived protein design jobs when idle. Compared to a linear approach, BinderFlow facilitates binder design through parallelisation and live monitoring, and by preventing superfluous calculation of more hits than can be experimentally screened. Overall, BinderFlow makes protein design more accessible, enabling its coexistence with other work in the same computing infrastructure.

We provide fully integrated code for deploying BinderFlow on SLURM-based [32] HPC clusters or smaller setups, such as individual workstations, alongside BFmonitor, a web-based application from which the user can interact with the pipeline, monitor the campaign in real time and prepare candidates for downstream applications.

## Design and implementation

### The BinderFlow pipeline

In BinderFlow, we have integrated backbone design, filtering of suboptimal backbones, sequence inference and score calculations in a continuous workflow, automating the input and output handling between each step (Fig 1A). As a result, binder design can be executed end-to-end in batches of a small number of designs per job.

A design campaign is split into multiple, parallel BinderFlow instances, each addressing a single batch of designs and independently executed on available GPUs (Fig 1B). Each BinderFlow instance executes the following scripts sequentially per GPU (Fig 1A):

 I) RFD.sh: runs RFD to produce *n* binder backbones.
 II) align_filtering.sh: replaces the cropped target chain with a complete version, providing more context for the sequence assignment and scoring algorithms. To avoid subsequent waste of computational resources, it also filters out designs with steric clashes and backbones composed of a single, long helix or a single hairpin, which are difficult to produce experimentally.
III) pMPNN.sh: runs pMPNN to assign sequences to the binder backbone in the context of the target.
IV) scoring.sh: predicts the binder-target complex structure using a modified version of AF2IG [24–26] to obtain the per-residue predicted Local Distance Difference Test (pLDDT) and Predicted Aligned Error (PAE) scores. Then, it calculates physics-based metrics using PyRosetta [33] to further filter and characterise the designs (e.g., shape complementarity [34] or number of unsatisfied hydrogen bonds [35]). See the Methods section for more details on scores and their calculation.

Before finishing, every BinderFlow instance appends the scores associated with each design to a.*csv* file for live monitoring of the campaign (Fig 1A) and evaluates the number of designs that meet the *in silico* conditions for a binder to be considered a hit (e.g., PAE_interaction < 10, pLDDT_binder > 80). This workflow is then repeated until the desired number of hits is obtained. This architecture is fully modular, so each step can be adapted to different software that performs similar functions, and further steps can be added or substituted as new tools become available.

The end-to-end architecture of BinderFlow instances allows independent execution and, thus, their parallelisation across different GPUs (Fig 1B). Additionally, BinderFlow jobs can be submitted with low priority to the queuing system, ensuring they are executed only when GPU nodes would otherwise remain idle. This facilitates the simultaneous execution of multiple campaigns and the parallelisation of multiple GPUs within a single campaign, reducing HPC queue clogging and improving the efficiency of shared computational resources. Overall, this workflow makes binder design more accessible to researchers with access to GPU infrastructure, but not primarily focused on protein design, such as structural biology laboratories.

### BinderFlow input and output

To run BinderFlow, the user defines an input structure – usually cropped to the region of interest to reduce computing times during the RFD steps – and a template structure with a more complete version of the protein, which provides more context for the sequence assignment and scoring steps. Both structures must be in.pdb format. The campaign information is provided to BinderFlow through a JSON file, in which the user must detail the path to the input and template structures, the desired range of binder sizes, and the number of parallel jobs, among other parameters. A detailed description and a template input file are available at https://github.com/cryoEM-CNIO/BinderFlow.

To facilitate the results interpretation, BinderFlow stores campaign results metrics in Scoring_Stats.csv, including both AF2IG and PyRosetta scorings of the designs.

### Real-time monitoring

**Fig 2:**
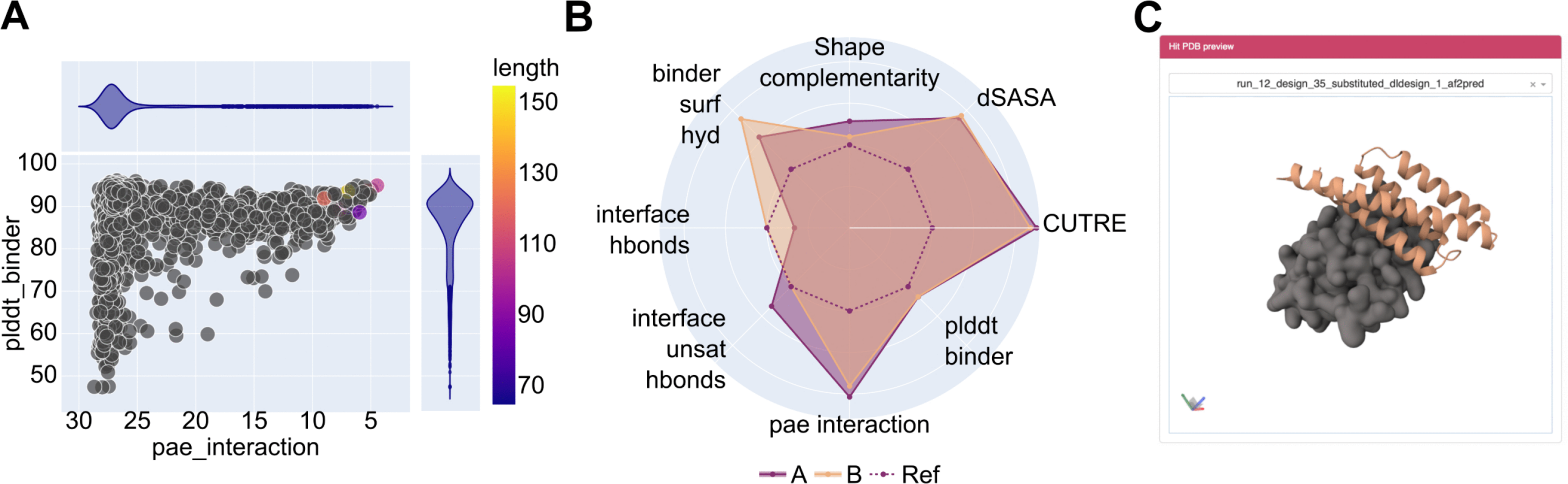
The BFmonitor web-based dashboard. BFmonitor provides three tools to follow a design campaign in real. **(A)** A scatter plot that updates in real time, providing information about any two of the variables calculated by scoring.sh. Each data point corresponds to an individual binder, and only those that fulfil all in silico criteria get colored. **(B)** A radar plot enables pairwise comparison of all scores calculated for any two binders, providing visual information on how each metric compares between the two candidates. **(C)** An interactive 3D viewer allows to inspect the backbones of in silico hits (yellow cartoon) interactively in the context of the target protein (grey surface) in real time.

Another advantage of BinderFlow’s end-to-end architecture is that it enables real-time monitoring of design campaigns as scores are calculated in small batches. To facilitate this, we developed BFmonitor, a web-based dashboard that includes tools to monitor campaigns, evaluate designs, and select hits for DNA synthesis (Figs 2, S1).

The first tool is Live Watcher, an interactive graphical summary of the design campaign. Within this interface, the user can set thresholds for all parameters calculated by AF2IG and PyRosetta to define when binders are considered hits and filter them. It contains a scatter plot, in which any pair of these parameters can be represented, providing an overview of the project and identifying correlations between them (Figs 2A, S1A). It also includes a radar plot for pairwise comparisons of designs, showing all calculated parameters normalised by the value set for the mentioned thresholds (Figs 2B, S1A). These metrics can be used to further filter hits, as recent studies have shown that PyRosetta-derived scores are effective for hit selection when combined with AF2IG confidence metrics [29]. However, the relevance of each metric is highly target-dependent. While default thresholds are provided for all scoring metrics, we recommend adjusting these values for each specific case. Detailed information on the metrics, their thresholds, and implementation is available in the GitHub repository: https://github.com/cryoEM-CNIO/BinderFlow.

The second tab, Pipeline tracking, details the progress of each instance. The last tab, Extraction (Figs 2C, S1B), allows the user to preview the structure of the binder-target complex for any design that passes the thresholds. In this window, the user can extract the hits structures as.pdb files and their sequences in.fasta format. In addition, we include a reverse translation tool based on CodonTransformer [36], which returns DNA coding sequences for the selected binders. These can be flanked by custom 5’ and 3’ sequences to facilitate cloning and downstream applications, and can be directly used for DNA synthesis orders.

## Results

### BinderFlow modularity enables binder refining strategies

Once an artificial or natural binder has been validated experimentally, it might still not meet desired biochemical criteria (e.g., affinity, specificity, solubility). Using protein design tools, it is possible to enhance their properties by further exploring the structural and sequence spaces. To improve previously identified binders, we have implemented two refining strategies into our pipeline: Partial Diffusion [22] and Sequence Diversity.

**Fig 3:**
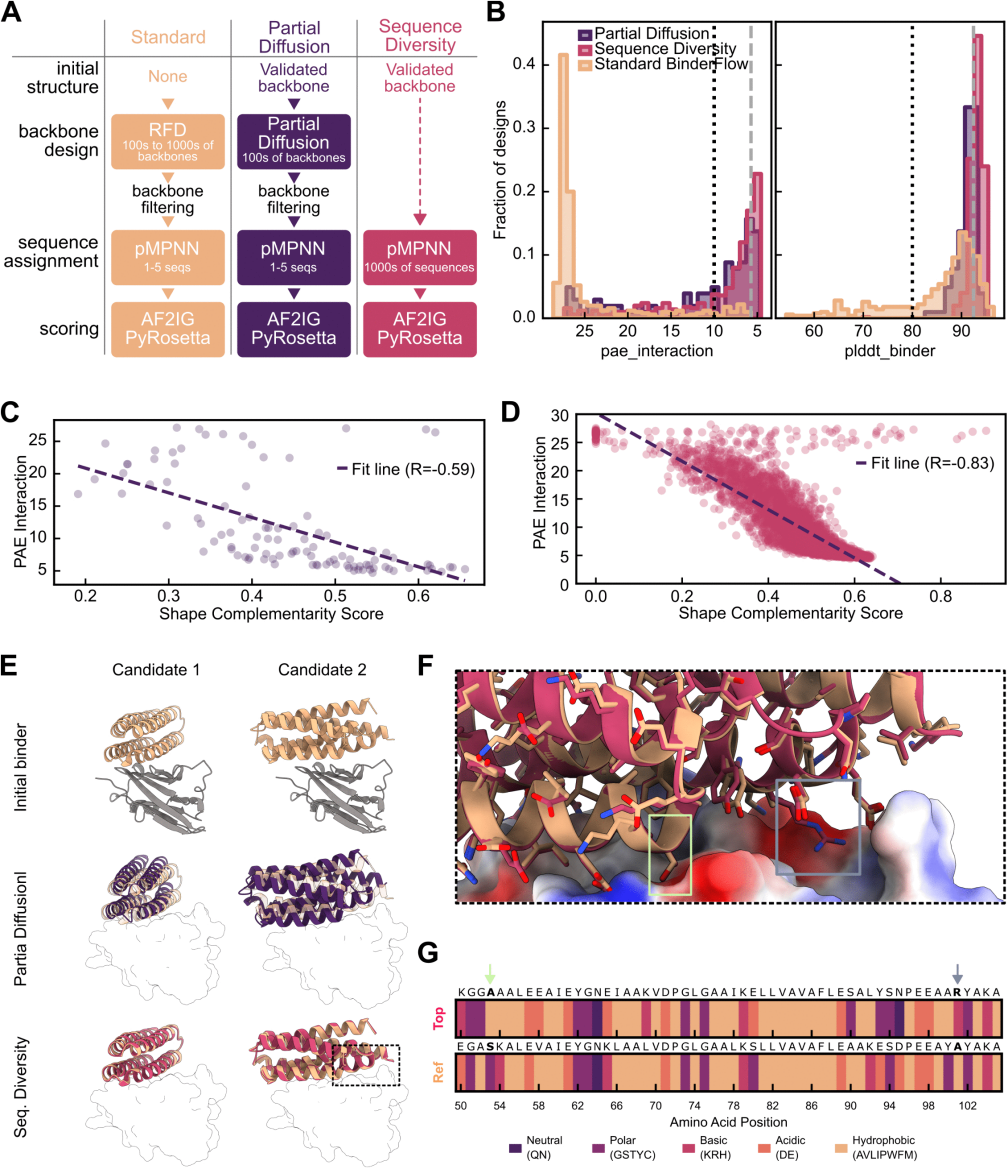
Alternative pipelines for binder refinement strategies: FastRelax and Sequence Diversity. **(A)** Comparison between the standard BinderFlow pipeline and proposed alternative strategies for binder refinement. The dashed arrow indicates skipping the backbone generation step. **(B)** Comparison of PAE interaction and binder pLDDT score distributions resulting from the standard BinderFlow pipeline, Partial Diffusion and Sequence Diversity. The grey, dashed lines indicate the pae_interaction and plddt_binder scores of the design used to initialize Partial Diffusion and Sequence Diversity runs, obtained from the standard BinderFlow pipeline. The black, dotted lines indicate typical thresholds to consider a binder a hit, PAE_interaction < 10 and pLDDT_binder > 80. **(C)** Correlation between PAE_interaction scores and Shape Complementarity scores for designs resulting from the Partial Diffusion run. **(D)** Correlation between PAE_interaction scores and Shape Complementarity scores for designs resulting from the Sequence Diversity run. **(E)** Structural alignments between candidates selected for *in silico* refinement (yellow) and example hits from Partial Diffusion (purple) and Sequence Diversity (red). The target, PDL1, is shown as a grey cartoon or as a silhouette. The first row displays the initial structure of the candidates, while the second and third rows show backbone structural variation for each candidate after the respective refinement strategy. **(F)** Predictions of the input reference (yellow, Ref) and the top hit according to the PAE interaction (red, Top), with PDL1 shown as an electrostatic surface. **(G)**_Residue_ identity changes introduced during Sequence Diversity shown on a sequence alignment of the interacting helix (residues 50–105) between Ref and Top. Two residue changes are highlighted in lime (S > A) and grey **(A > R)**.

Partial Diffusion employs a method previously described by Vazquez-Torres *et al.* [21], in which, instead of random coordinates, the atomic positions of a validated backbone are used as the starting point. These positions are disturbed by adding white noise —typically a fraction of the noise level used for RFD— and then “denoised” to explore the nearby structural space, where new energy minima might be found. This step replaces regular RFD backbone inference, and the rest of the process follows the same steps as the pipeline described above (Fig 3A).

The Sequence Diversity strategy uses pMPNN to sample the sequence space. pMPNN designs new sequences that likely fold into the previously specified backbone, while integrating the context of the target interface. Providing a validated backbone as input, we massively run pMPNN to obtain many sequences that are predicted to fold into the same structure (Fig 3A). This strategy fully skips the backbone generation step, making it less computationally expensive than Partial Diffusion.

Compared to *de novo* backbone design, the distribution of *in silico* metrics after Partial Diffusion is skewed towards what is considered a hit (Figs 3B, S2A). The improvement in metrics is likely due to a better fit of the backbones to the target surface (Fig 3C). The distribution of designs from Sequence Diversity changed similarly (Figs 3B, S2A). The increase in success rate correlates with the shape complementarity of the binder sequence to the target (Fig 3D), but not with sequence similarity to the original sequence (S2B,S2C Fig). This demonstrates that Sequence Diversity gently explores the structural space around the input structure, as different sequences induce slight structural adjustments to accommodate the different amino acids while maintaining the overall fold (Fig 3E-3G). Further structural exploration within Sequence Diversity could be achieved by performing pMPNN with FastRelax, a protocol that slightly modifies the binder backbone by energy minimisation [24,37]. However, we have not observed an increase in *in silico* hits obtained using FastRelax on RFD-calculated backbones (S3 Fig), as others have noted [22].

It is important to note that the performance of each design strategy varies across proteins, as different backbones require different degrees of exploration of the sequence and structural landscape to optimise binding to their targeted surface (S2A Fig). BinderFlow enables the efficient exploration and combination of multiple design strategies, facilitating the design process.

### BinderFlow benchmarking

**Fig 4:**
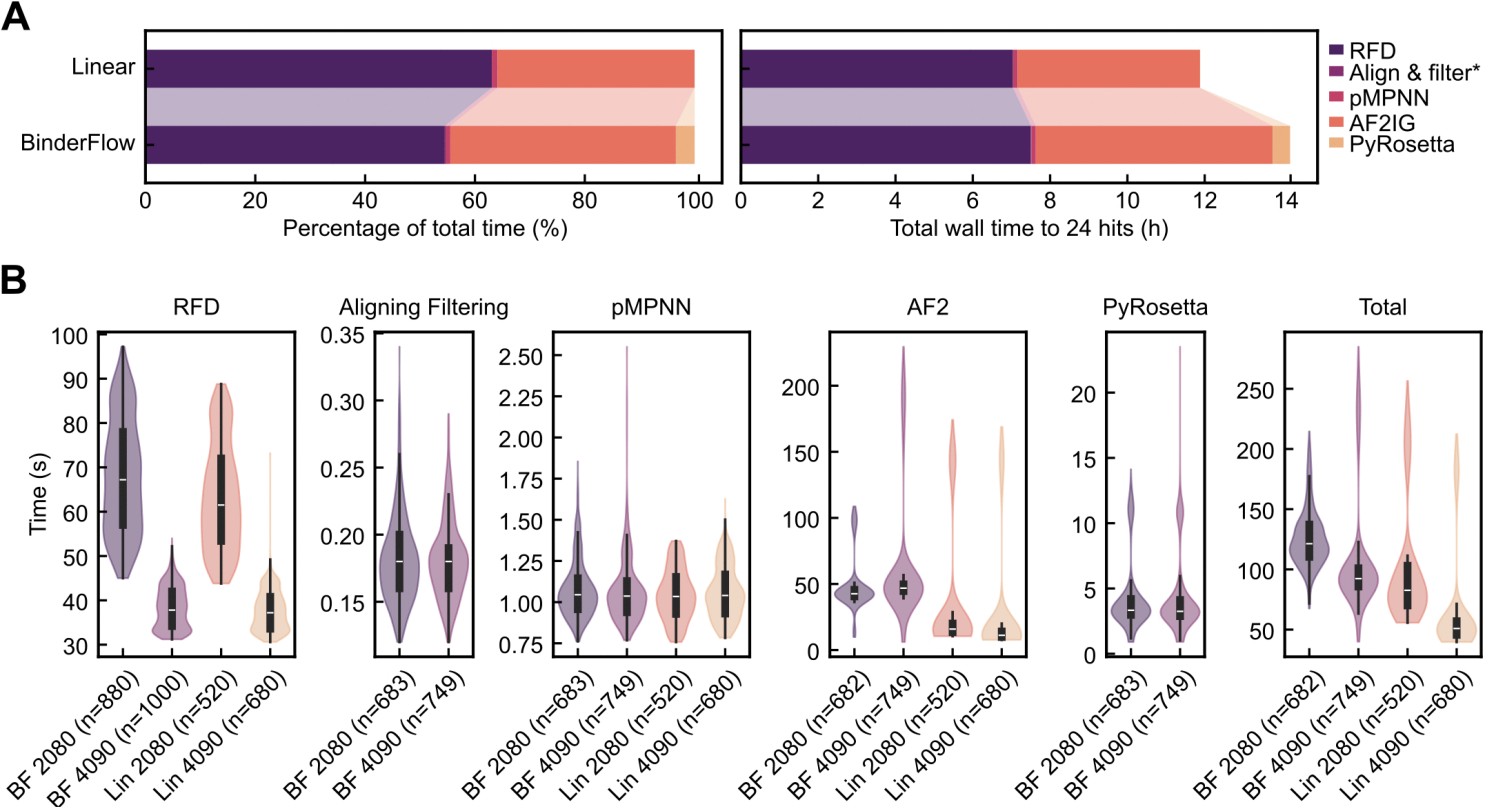
Benchmarking of BinderFlow. **(A)** Comparison of time spent per step using a linear binder design pipeline and the BinderFlow architecture. Left: normalized times and proportion of the run length allocated to each step. Right: comparison of the total wall times required to obtain 24 hits. *Align & filter times are too small to be noticed in the bar plot (S1 Fig). **(B)** Comparison of wall time per pipeline step between the linear approach (Lin) and BinderFlow (BF). The same campaigns were executed on RTX4090 and RTX2080 GPUs. The number of designs corresponding to each step is indicated on the x axis. “Aligning Filtering” and “PyRosetta” steps are only performed by BinderFlow.

To characterise how the batch-based architecture of BinderFlow affects the performance of the pipeline, we launched a binder design campaign against Programmed Death-Ligand (PDL1), a protein often employed as a benchmark for binder generation [17,22,38]. The designs ranged from 65 to 155 residues, using both BinderFlow and the linear pipeline (see Methods for details). The efficiencies of RFD and pMPNN remained largely unchanged. The linear pipeline averages 63.5 s and 1.0 s per design, respectively, compared to 68.0 s and 1.1 s per design in BinderFlow (Fig 4A,4B). Initially, the total processing time per binder increased due to the AF2IG scoring step, which required 273% more time when utilising the BinderFlow structure (134.3 s using BinderFlow versus 36.1 s using the linear pipeline). Splitting large jobs into smaller batches required restarting calculations for each batch, which came at the cost of losing intermediate results stored in memory. Upon inspecting the AF2IG code [24], we observed that each prediction required an intermediate calculation that is computed once for a given design length and then recycled for all designs with the same number of amino acids, reducing scoring times by 10-fold (S4A Fig). By splitting the design campaign into batches, this calculation took place hundreds of times instead of once per design length, significantly slowing down the process. We alleviated this inefficiency by writing the matrices resulting from those calculations to disk and reusing them across batches. Thus, the modified version of AF2IG requires only 51.3 s per binder when executed as part of BinderFlow, reducing the total time difference between the linear and BinderFlow pipelines to 24% (Figs 4A,4B, S4B). Importantly, the time lost per binder due to slower scoring was partially offset by automatic backbone filtering after RFD (Fig 1A), which avoids wasting time on sequence inference and scoring of suboptimal backbones. It took BinderFlow 14.2 h of total wall time to obtain 24 hits, compared with 12 h for the linear pipeline. Thus, the difference in efficiency is reduced from 24% to 18% (Figs 4A,4B, S4B). Note that this comparison assumes the best-case scenario for the linear pipeline, where the run’s efficiency is known, thereby avoiding the waste of computation on more hits than required and the time spent re-running the pipeline due to underestimation. In real-case scenarios, BinderFlow’s live monitoring of the hit rate prevents superfluous calculation of more hits that can be experimentally screened, reducing the efficiency gap even further.

### Availability and future directions

The introduction of generative models has significantly streamlined the process of binder design. Tasks that previously required substantial expertise in protein engineering can now be executed efficiently using these computational tools. As a result, the main bottleneck has shifted from technical knowledge in protein design to biological insights for selecting relevant targets and robust validation strategies. Yet, while many laboratories have the necessary biological expertise, they often lack the computational infrastructure and expertise required to deploy large-scale binder design projects. Moreover, repurposing existing computational resources to support protein design is not always feasible.

Here, we introduce BinderFlow, a pipeline designed to democratise and facilitate *de novo* protein binder design for both non-experts and experts. BinderFlow divides otherwise large protein design campaigns into batches, enabling their coexistence with other resource-intensive activities in the same computing infrastructure. It features a parallelizable architecture and automates most tasks that usually require human intervention, such as handling input and output or rejecting suboptimal candidate backbones. Its batch-based design enables live monitoring of campaigns, enabling real-time estimation of campaign efficiency. Moreover, computational resources are allocated efficiently by avoiding superfluous calculations after the desired number of hits is obtained.

We provide BinderFlow as a ready-to-use implementation for SLURM-based [32] systems, ranging from HPCs to individual workstations. Although the GPU time per binder increases using this pipeline compared to a linear workflow (Fig 4A), this difference can be alleviated by increasing the batch size and reducing the length range (S4A Fig), albeit at the cost of fewer monitoring updates.

Both BinderFlow and BFmonitor are available as open software, and free to download, adapt, and modify from GitHub (https://github.com/cryoEM-CNIO/BinderFlow). The repository includes instructions for installation and usage of both tools, along with descriptions of all input parameters to facilitate their use. The PDL1 target structure used to benchmark BinderFlow in this publication is also openly available from AFDB (AFDB AF-Q9NZQ7-F1-v4).

Anticipating the quick pace at which new algorithms for all steps (backbone design, sequence inference and scoring of candidates) become available, we designed BinderFlow as a modular pipeline, streamlining the incorporation of new software to give users the option to use their preferred tool for each step. This modularity also enables flexible adaptation of the design workflow itself. We illustrate this by providing two ready-to-use strategies for binder refinement that explore both the structure and sequence space: Partial Diffusion and Sequence Diversity. Our *in silico* binder refinement results suggest that there is room for alternative protocols to improve binder affinity and specificity; thus, we expect more strategies to be implemented that will increase the design success rate. Nonetheless, the relationship between computational confidence metrics and experimental binder success rates remains poorly understood. Advancing the experimental efficiency of protein design will require new, more predictive metrics that integrate quantitative affinity or phenotypic data collected by coherent experimental approaches, prediction models, and biophysical scoring functions.

## Methods

### Benchmarking

For all benchmarking campaigns, consumer graphics cards (RTX2080Ti or RTX4090Ti) were employed as indicated, as they represent standard and affordable cards commonly used in laboratories employing GPU-based routines with relatively low memory requirements, such as structural biology groups.

For the BinderFlow benchmarking campaigns, a trimmed version of PDL1 (residues 18–132, AFDB AF-Q9NZQ7-F1-v4) was used as target to decrease computation times. The selection of this protein is due to its use as a benchmark in previous publications with different binder generation models [17,22,38]. The structure was renumbered to start in chain B and residue 1018 to prevent indexing issues with AF2IG. Residues 1054 and 1068 (I54 and V68 in untrimmed PDL1, respectively) were selected as hotspots for binder generation. Binders of lengths comprising 65–155 residues were designed using RFD, with default parameters (complex_base checkpoint, 50 noise steps). 10-backbone batches were designed per available GPU.

Binder backbones were selected attending to: [1] the presence of steric clashes (defined as having at least one atom within 0.5 Å from the target structure), and [2] their tertiary structure, filtering out designs predicted to fold as hairpins or long, single helices using DSSP [39]. One sequence per backbone was generated using pMPNN without FastRelax optimization [24], and binder-target complexes were used as input for AF2IG scoring and PyRosetta analysis to extract a series of biophysical metrics (see below for more details). The campaigns were run until obtaining at least 48 *in silico* hits.

For the campaigns following the linear pipeline, the same input and hotspots were used. For the backbone generation step, 520 and 680 designs were generated using RFD with the RTX 2080 Ti and RTX 4090 Ti GPUs, respectively, based on our previous experience with PDL1 binder design hit rate. Next, we generated one sequence per design using pMPNN without FastRelax and, lastly, they were scored using AF2IG.

A binder was considered a hit following the description by Bennet et al. [24] and followed by others [17,22,40,41] (pLDDT_binder > 80 and PAE_interaction < 10). Despite not using them for benchmarking purposes, we provide several different metrics obtained from PyRosetta-based scripts [33] and the AlphaFold2 scoring runs [25,26] that can be used for further filtering. PyRosetta metrics were calculated using InterfaceAnalyzer, including, difference in solvent accessible surface area (dSASA), shape complementarity (as described by Lawrence and Colman [34]) and the number of unsatisfied and satisfied hydrogen bonds (which should be minimized and maximized, respectively). We also derived metrics from AlphaFold2 confidence scores, and provide ipSAE [28] and CUTRE (Coherent Unbiased meTric for bindeR dEsign).

CUTRE computes the average PAE value of the interface residues (those with at least one atom ≤10 Å away the opposite chain), weighting their contribution by its pLDDT. Formally:


$$\text{CUTRE}=\frac{1}{2N_{T}N_{B}}\left(\sum {\mathrm{\backslash nolimits}}_{i\in T}\sum {\mathrm{\backslash nolimits}}_{j\in B}\frac{{\text{PAE}}_{ij}}{{\text{pLDDT}}_{i}/100}+\sum {\mathrm{\backslash nolimits}}_{j\in B}\sum {\mathrm{\backslash nolimits}}_{i\in T}\frac{{\text{PAE}}_{ji}}{{\text{pLDDT}}_{j}/100}\right)$$ (1)

where T and B respectively denote the set of target and binder interface residues, $N_{T}$ and $N_{B}$ the number of residues in each set, ${PAE}_{ij}$ the PAE of residue j when the structure is aligned at residue i, and ${pLDDT}_{i}$ is the pLDDT of residue i. Being fundamentally a weighted PAE, lower values represent a more confident prediction. This metric overcomes a known limitation of the PAE_interaction, which is the calculation of the PAE in proteins with flexible or badly predicted regions [28].

### Sequence diversity and partial diffusion runs

Partial Diffusion campaigns following BinderFlow architecture were performed only in Nvidia RTX2080Ti GPU cards, as we did not observe a significant change in efficiency with respect to the RTX4090Ti GPU cards. Five independent candidate binders were selected from the benchmarking campaigns as input for both Sequence Diversity and Partial Diffusion, based on their metrics and the presence of viable, diverse folds (S2 Fig). For Partial Diffusion campaigns, default noise settings were employed (Complex_base as checkpoint, 20 noising steps, noise scale of 1 for both translations and rotations). 10-backbone batches were designed per available GPU. Backbones were filtered, assigned a single sequence using pMPNN without FastRelax and scored by AF2IG and PyRosetta as indicated above. The campaign continued until at least 48 hits were obtained.

For Sequence Diversity campaigns, between 5000 and 5400 sequences were designed in batches of 200 sequences per GPU using pMPNN without the Fast Relax protocol. The designs were then scored using AF2IG and PyRosetta. The same thresholds for hits as in previous campaigns were used.

For Sequence Diversity with Fast Relax campaigns, 200 sequences were designed per input structure (same as in Partial Diffusion and Sequence Diversity campaigns), in batches of 1 sequence with 1 FastRelax cycle. Designs were scored as indicated above.

## Supporting information

S1 Fig BFmonitor overview. A) Live Watcher allows to monitor binder campaigns in real time and filter hits using user-defined thresholds for all the scores calculated by AF2IG and PyRosetta. These thresholds can be adjusted with the sliders on the left panel of the dashboard. B) Extraction allows for visual inspection of the backbone of the selected hits and extraction of their structures as PDBs or their sequences as FASTAs. It also contains tools for codon-optimized, reverse translation of the protein sequences, yielding ready-to-order DNA coding sequences.
(TIF)

S2 Fig Efficiency comparison between the standard, Partial Diffusion and Sequence Diversity pipelines. A) Comparison of PAE interaction and binder pLDDT distributions resulting from campaigns run using the standard BinderFlow pipeline, Partial Diffusion and Sequence Diversity. Each row represents runs initiated with a different in silico hit from the same Standard BinderFlow run. The grey dashed lines indicate the PAE interaction and pLDDT binder scores of the design obtained from the standard BinderFlow pipeline used as backbone to initialise Partial Diffusion and Sequence Diversity runs. The black, dotted lines indicate typical thresholds to consider a binder a hit, i.e., PAE_interaction < 10, pLDDT_binder > 80. B) Correlation between PAE_interaction scores and BLOSUM62 scores, a measure of sequence similarity, calculated between binders resulting from the Sequence Diversity run and the original candidate. C) Sequence alignment of binders obtained from a Sequence Diversity run. Top 20: the 20 highest-scoring binders ordered by PAE_interaction. Ref: the sequence of the binder used as template for initiating the Sequence Diversity run. Bottom 20: the 20 lower-scoring binders ordered by PAE_interaction.
(TIFF)

S3 Fig FastRelax does not consistently improve Sequence Diversity in silico hit rates. A) Comparison of PAE interaction and binder pLDDT distributions resulting from campaigns run using Sequence Diversity with and without the FastRelax protocol. Each row represents runs initiated with a different in silico hit from the same Standard BinderFlow run. The black, dotted lines indicate typical thresholds to consider a binder a hit, i.e., PAE_interaction < 10, pLDDT_binder > 80. B) Comparison of wall time per step in Sequence Diversity using, or not, the FastRelax. The number of designs corresponding to each step is indicated on the x-axis. C) Comparison of in silico hit rates for five different Sequence Diversity runs with and without the FastRelax protocol. The raw count of hits for each candidate is indicated in parentheses.
(TIFF)

S4 Fig Benchmarking of BinderFlow. A) Order of prediction impacts AF2IG scoring wall times. Length Occurrence stands for how many designs of that same length have been predicted in that same batch. Times extracted from the linear pipeline. B) Table summarising the time spent per binder and design strategy in each step, comparing GPU models and binder generation pipelines.
(TIFF)
