# Supplementary figures and images for: Automated and modular protein binder design with BinderFlow
Source: PLoS Comput Biol. 2025 Nov 21;21(11):e1013747. doi: 10.1371/journal.pcbi.1013747 (PMC12668606; doi:10.1371/journal.pcbi.1013747)

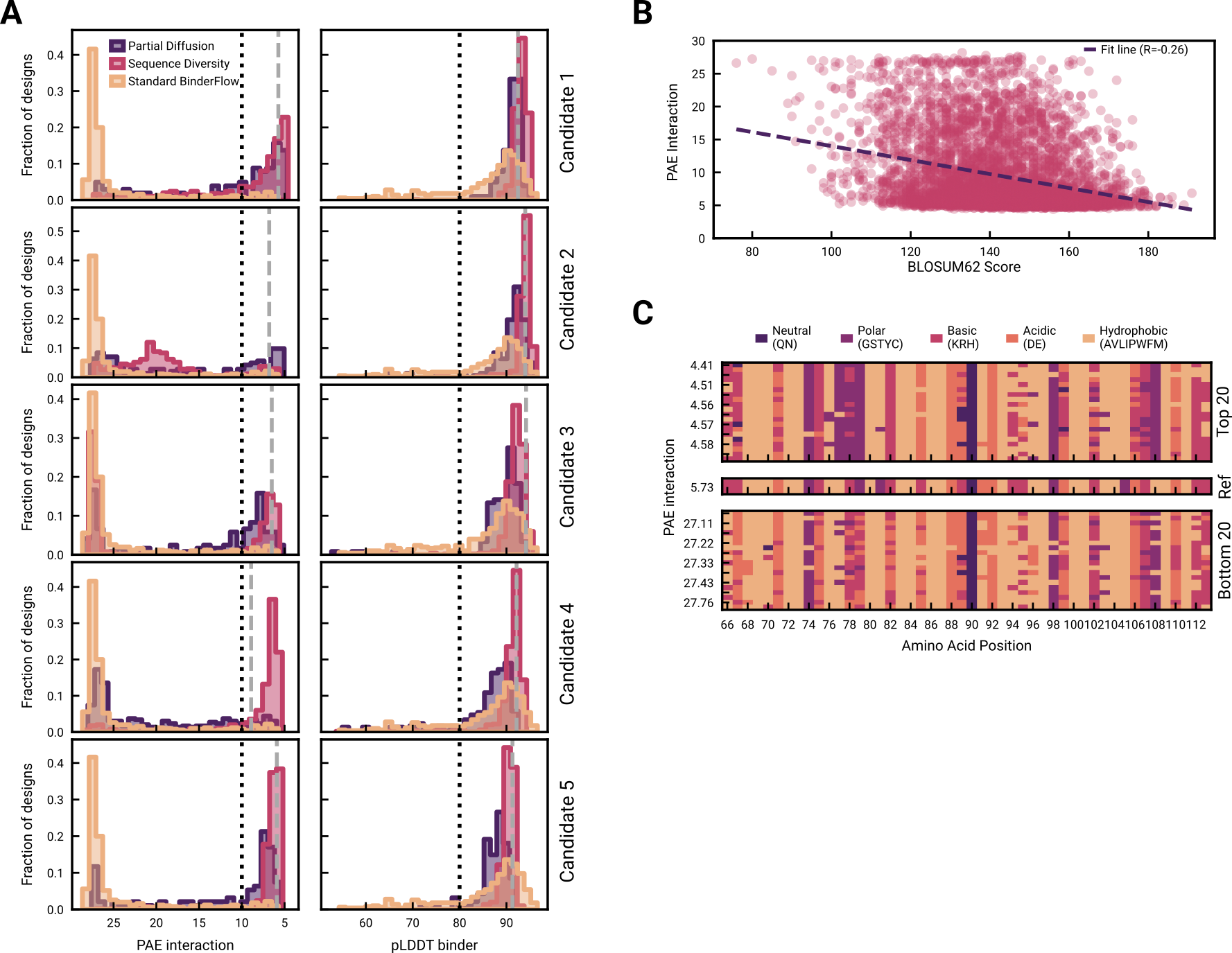

Supplement: S2 Fig — A) Comparison of PAE interaction and binder pLDDT distributions resulting from campaigns run using the standard BinderFlow pipeline, Partial Diffusion and Sequence Diversity. Each row represents runs initiated with a different in silico hit from the same Standard BinderFlow run. The grey dashed lines indicate the PAE interaction and pLDDT binder scores of the design obtained from the standard BinderFlow pipeline used as backbone to initialise Partial Diffusion and Sequence Diversity runs. The black, dotted lines indicate typical thresholds to consider a binder a hit, i.e., PAE_interaction < 10, pLDDT_binder > 80. B) Correlation between PAE_interaction scores and BLOSUM62 scores, a measure of sequence similarity, calculated between binders resulting from the Sequence Diversity run and the original candidate. C) Sequence alignment of binders obtained from a Sequence Diversity run. Top 20: the 20 highest-scoring binders ordered by PAE_interaction. Ref: the sequence of the binder used as template for initiating the Sequence Diversity run. Bottom 20: the 20 lower-scoring binders ordered by PAE_interaction. (TIFF) [file pcbi.1013747.s002.tiff]

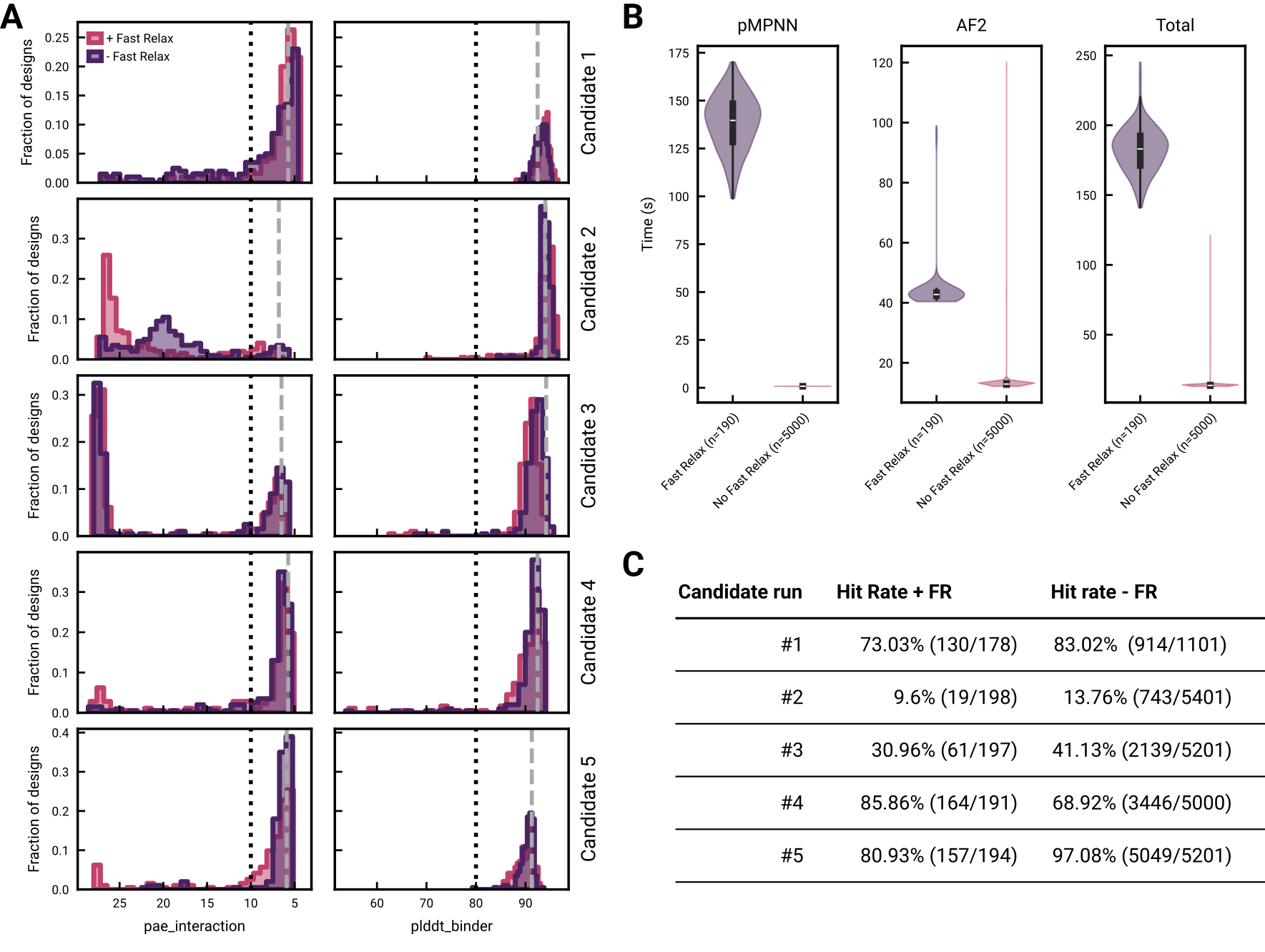

Supplement: S3 Fig — A) Comparison of PAE interaction and binder pLDDT distributions resulting from campaigns run using Sequence Diversity with and without the FastRelax protocol. Each row represents runs initiated with a different in silico hit from the same Standard BinderFlow run. The black, dotted lines indicate typical thresholds to consider a binder a hit, i.e., PAE_interaction < 10, pLDDT_binder > 80. B) Comparison of wall time per step in Sequence Diversity using, or not, the FastRelax. The number of designs corresponding to each step is indicated on the x-axis. C) Comparison of in silico hit rates for five different Sequence Diversity runs with and without the FastRelax protocol. The raw count of hits for each candidate is indicated in parentheses. (TIFF) [file pcbi.1013747.s003.tiff]

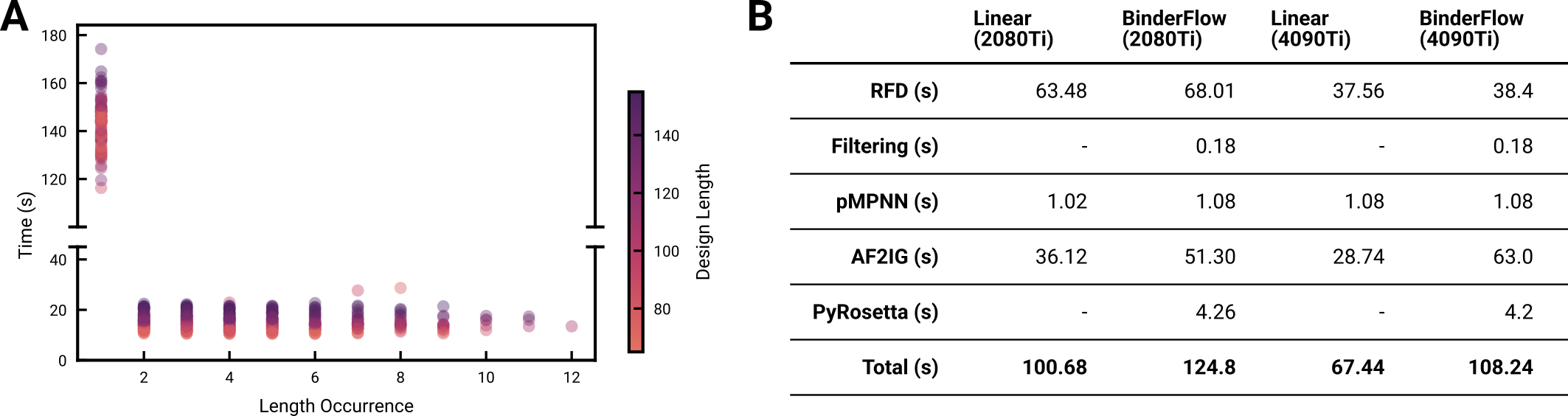

Supplement: S4 Fig — A) Order of prediction impacts AF2IG scoring wall times. Length Occurrence stands for how many designs of that same length have been predicted in that same batch. Times extracted from the linear pipeline. B) Table summarising the time spent per binder and design strategy in each step, comparing GPU models and binder generation pipelines. (TIFF) [file pcbi.1013747.s004.tiff]
